# Left‐Side Versus Right‐Side Lateral Tilt During Maternal CPR: Effects on Compression Quality and Rescuer Fatigue

**DOI:** 10.1155/emmi/3607809

**Published:** 2026-02-26

**Authors:** Chia-Lung Kao, Jui-Yi Tsou, Ming‐Yuan Hong, Chih-Jan Chang, Fong-Chin Su, Chih-Hsien Chi

**Affiliations:** ^1^ Department of Emergency Medicine, National Cheng Kung University Hospital, College of Medicine, National Cheng Kung University, Tainan, Taiwan, ncku.edu.tw; ^2^ Department of Physical Therapy, Fooyin University, Kaohsiung, Taiwan, fy.edu.tw; ^3^ Department of Biomedical Engineering, National Cheng Kung University, Tainan, Taiwan, ncku.edu.tw; ^4^ Medical Device Innovation Center, National Cheng Kung University, Tainan, Taiwan, ncku.edu.tw

**Keywords:** biomechanical study, left lateral tilt chest compression, manikin study, maternal cardiac arrest, pregnant CPR

## Abstract

**Background:**

Maternal cardiac arrest presents unique challenges due to physiological changes in pregnancy. Left lateral tilt (LLT) is commonly recommended to relieve aortocaval compression, but its impact on chest compression quality remains unclear.

**Objectives:**

This study evaluates chest compressions performed in the LLT position from both the right and left sides to determine if they meet high quality cardiopulmonary resuscitation standards.

**Methods:**

This randomized crossover study included 44 healthcare providers performing two‐minute chest compressions’ sessions on a manikin in the LLT position from both right and left sides. Compression depth, rate, recoil, force distribution, rescuer fatigue, and physiological parameters were analyzed.

**Results:**

Both approaches maintained adequate compression rates, but left‐side LLT chest compressions achieved better depth (41.23 ± 9.11 mm vs. 35.50 ± 9.54 mm, *p* < 0.001) and complete recoil (67.05 ± 39.05% vs. 38.39 ± 34.23%, *p* < 0.001). Left‐side LLT chest compressions also generated higher peak force and lower residual release force. Right‐side LLT chest compressions were associated with greater rescuer fatigue and instability.

**Conclusion:**

Left‐side LLT chest compression provides superior compression depth and recoil compared with right‐side LLT chest compression. However, neither method consistently meets high quality cardiopulmonary resuscitation standards. These findings support the 2015 AHA guideline preference for manual uterine displacement over LLT chest compression. Further research is needed to optimize maternal cardiac arrest management.

## 1. Introduction

Resuscitation during maternal cardiac arrest presents a significant clinical challenge [1]. Cardiac arrest is a critical emergency resulting from major causes of maternal mortality such as hemorrhage, hypertensive disorders, and sepsis [2–4]. The reported incidence of maternal cardiac arrest ranges from 1 in 12,000 to 1 in 36,000 pregnancies, with survival rate varying from 15% to 54% [5–11]. Common causes of cardiac arrest and maternal death during pregnancy include anesthesia complications, cardiovascular disease, obstetric hemorrhage, thromboembolism, uterine atony, and eclampsia [12–15].

Pregnancy includes a lot of physiological changes, including increased blood volume and preload, reduced systemic and peripheral vascular resistance, elevated heart rate (HR) and cardiac output, increased tidal volume and respiratory depth, a rise in red blood cell mass accompanied by relative anemia, and a hypercoagulable state [16–18]. Aortocaval compression becomes a critical concern after 20 weeks of gestation, as the gravid uterus can compress the inferior vena cava (IVC), leading to reduced venous return, diminished preload, decreased cardiac output, and compromised perfusion [16–19].

The use of the left lateral tilt (LLT) to relieve IVC compression was first described in 1940s by McLennan and Crawford et al. More recent studies have demonstrated that a 15 degree of LLT provides little relief of IVC compression, whereas a tilt of at least 30° is required to achieve significant decompression [20–23].

High quality cardiopulmonary resuscitation (HQCPR) can increase survival after cardiac arrest, with chest compression quality serving as a major component of HQCPR.

HQCPR enhances brain perfusion and improves overall outcomes in patients experiencing cardiac arrest. High quality external chest compressions (ECCs) are characterized by correct hand placement, a compression rate of 100–120 per minute, a depth of 5–6 cm, and complete chest recoil [24, 25]. In addition, the distribution of force during ECC may help identify the optimal compression site to minimize injuries, including rib and sternal fractures, spinal trauma, splenic rupture, or liver lacerations [26–29].

The American Heart Association (AHA) mentions the topic of maternal cardiac arrest since 1992. Since then, the use of LLT or manual left lateral uterine displacement during CPR in pregnant patients has been recommended and 2010 AHA guideline also recommends the use of LLT or manual left lateral uterine displacement [30]. ECC can be performed on pregnancy women with LLT on a wedge or on the human wedge position [31, 32]. The human wedge position involves a rescuer kneeling on the floor and sitting on their heels, allowing the patient’s back to place on the rescuer’s thighs [32]. When performing LLT ECC on a wedge, ECC can be administered from either the patient’s right or left side. In contrast, under the human wedge technique, ECC is typically delivered from the patient’s right side only.

However, the 2015 AHA guidelines recommend manual uterine displacement rather than tilt due to LLT ECC being significantly less effective than ECC in the usual supine position, but this commendation does not have strong evidences (level of evidence C) [33–36].

Evidences regarding the quality of LLT ECC remain limited. Therefore, this study aimed to compare the force distribution and quality of LLT ECC performed from the right versus the left side and to determine whether LLT ECC can meet the criteria for HQCPR.

## 2. Methods

### 2.1. Setting and Design

This was a randomized crossover observational study. A pilot study involving 10 participants was conducted to calculate the required sample size by G∗Power [37]. The calculated effect size was 0.45. Based on a power of 80% and a significance level of 0.05, the minimum sample size required was 41 participants. To account for potential dropouts, a 10% increase was applied, yielding a final target enrollment of 45 participants. However, one participant was subsequently excluded from the analysis due to data loss caused by a recording failure.

All participants were experienced healthcare providers without any musculoskeletal or neurological injuries within 6 months. Chest compressions were performed according to the ACLS guidelines, targeting a depth of 5‐6 cm, a rate of 100–120 compressions per minute, and full chest recoil [24]. Informed consents approved by the Institutional Review Board were signed before the study.

**FIGURE 1:**
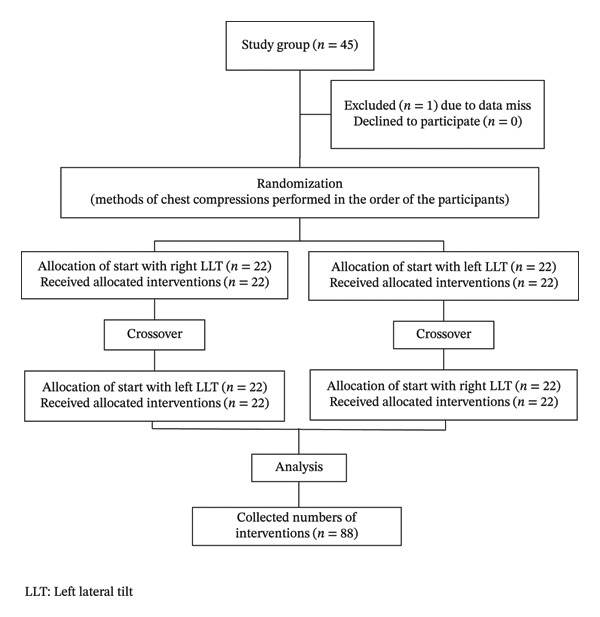
Flowchart of right‐side and left‐side LLT chest compressions.

ECC was performed on a Resusci Anne manikin positioned at a 30° LLT. Each participant completed 2 minutes of right‐side LLT ECC and 2 minutes of left‐side LLT ECC. These two methods were performed in random order, and adequate rest was given between the two sessions of ECC for more than 30 min. This was a one‐rescuer CPR model, and the participants performed ECC only without ventilation. Prior to data collection, all participants were allowed to practice ECC on the tilted manikin to ensure familiarity with the setup. To standardize technique, participants were instructed to place their right hand down during all compressions. The flowchart of this study protocol is shown in Figure 1.

### 2.2. Data Collection

Participants performed ECC on a Resusci Anne manikin (Laerdal Medical, Wappingers Falls, NY) positioned in a 30‐degree LLT. The manikin was connected to the Laerdal PC Skill Reporting System (QCPR), which continuously recorded compression quality parameters. These included hand position, compression depth, correct depth ratio, compression rate, correct rate ratio, and complete recoil ratio.

The Force Sensing Array system (*MatScan*, Pressure Measurement System Evolution Based, Tekscan Inc., South Boston, MA, USA) was used to measure the pressure, force, and the stability of the hand. *MatScan* used the piezoelectric principle to measure the pressure value and had 44 rows and 52 columns with 2288 pressure sensors per cm^2^ and a spatial resolution of 1.4 sensors per cm^2^. The sensor mat is 0.1 mm thick and can record the data at a sampling frequency of 30 Hz. The flexible pressure pad was securely affixed to the surface of the manikin to ensure consistent measurement during compressions. This system has demonstrated excellent reliability, with intraclass correlation coefficients ranging from 0.98 to 0.99, and has been widely used in medical pressure measurements [26, 38, 39].

Systolic blood pressure (SBP), diastolic blood pressure (DBP), and HR were recorded immediately before and after each ECC session. Participant fatigue and discomfort were assessed using a modified Borg scale ranging from 1 to 10 [40].

### 2.3. Data Analysis

Statistical analyses were performed using SPSS Statistics, Version 20.0 (IBM Corp., Armonk, NY, USA). Paired *t* tests were used to compare outcomes between the two conditions. A two‐tailed *p* value of < 0.05 was considered statistically significant.

## 3. Results

A total of 44 participants who included 22 females and 22 males were enrolled in the study. The age ranged from 24 to 52 years old, with a mean age of 32.3 ± 6.8 years. Among them, 14 were medical physicians, 29 were registered nurses, and 1 was an emergency medical technician. All participants held a valid ACLS certification.

### 3.1. Proportion of HQCPR in LLT Chest Compressions (Table 1)

As shown in Table 1, the compression depth achieved during right‐side LLT ECC was predominantly between 2 and 5 cm, with only 2 of 44 participants (4.5%) reaching the recommended depth of 5‐6 cm, as specified by ACLS guidelines. In comparison, left‐side LLT ECC yielded slightly better performance, with most compressions ranging from 3 to 5 cm, and 7 participants (15.9%) achieving the target depth. The majority of participants were able to maintain compression rates within the recommended range of 100–120 compressions per minute for both positions, the proportion of compressions with complete chest recoil. A greater proportion of left‐side LLT ECC sessions achieved complete recoil, whereas most right‐side compressions fell short of this standard.

**TABLE 1:** Proportion of HQCPR in LLT chest compressions.

Quality metric (*n* = 44)	Category	Right LLT (*n*, %)	Left LLT (*n*, %)
Compression depth	5–6 cm	2 (5%)	7 (18%)
	4–5 cm	13 (32%)	20 (51%)
	3–4 cm	16 (39%)	13 (33%)
	2–3 cm	12 (29%)	4 (10%)
	1–2 cm	1 (3%)	0 (0%)
Compression rate	100–120/min	38 (86%)	41 (93%)
	> 120/min	6 (14%)	3 (7%)
	< 100/min	0 (0%)	0 (0%)
Chest recoil	80%–100%	9 (20%)	25 (57%)
	60%–80%	4 (9%)	4 (9%)
	40%–60%	3 (7%)	3 (7%)
	20%–40%	11 (25%)	2 (5%)
	0%–20%	18 (41%)	10 (23%)

Abbreviation: LLT = left lateral tilt.

### 3.2. Comparison of ECC Quality between Right and Left LLT Positions (Table 2).

The performance of chest compressions in the right and left LLT positions is summarized in Table 2. There were no statistically significant differences between the two positions in terms of compression rate (113.68 ± 5.70 vs. 112.41 ± 6.06 compressions per minute, *p* = 0.120), correct rate ratio (77.45% ± 26.32% vs. 83.59% ± 21.21%, *p* = 0.136), or correct hand position ratio (96.55% ± 11.12% vs. 94.82% ± 17.84%, *p* = 0.412).

**TABLE 2:** ECC Quality between right and left LLT positions.

ECC performance	Right side (*n* = 44)	Left side (*n* = 44)	*P* value
	Mean ± SD	Mean ± SD	
ECC quality (%)	28.75 ± 26.07	50.27 ± 32.40	< 0.001^∗^
Correct hand position (%)	96.55 ± 11.12	94.82 ± 17.84	0.412
Compression depth (mm)	35.50 ± 9.54	41.23 ± 9.11	< 0.001^∗^
Correct depth (%)	11.25 ± 21.80	19.91 ± 31.53	0.034^∗^
Compression rate (per min)	113.68 ± 5.70	112.41 ± 6.06	0.12
Correct rate (%)	77.45 ± 26.32	83.59 ± 21.21	0.136
Complete recoil (%)	38.39 ± 34.23	67.05 ± 39.05	< 0.001^∗^

Abbreviations: ECC = external chest compression; LLT = left lateral tilt.

^∗^Statistical differences.

However, compressions performed from the right side demonstrated significantly lower overall ECC quality, as recorded by the QCPR system (28.75% ± 26.07% vs. 50.27% ± 32.40%, *p* < 0.001). The right‐side group also showed reduced compression depth (35.50 ± 9.54 mm vs. 41.23 ± 9.11 mm, *p* < 0.001), lower depth accuracy (11.25% ± 21.80% vs. 19.91% ± 31.53%, *p* = 0.034), and a significantly lower proportion of complete chest recoil (38.39% ± 34.23% vs. 67.05% ± 39.05%, *p* < 0.001).

Although both positions achieved compression rates consistent with ACLS guidelines, the left‐side LLT compressions demonstrated superior overall quality, primarily due to greater compression depth and more complete recoil. Nonetheless, the correct depth ratio in both groups remained suboptimal.

### 3.3. Forces Distribution (Table 3)

Table 3 summarizes the peak and release forces during chest compressions in both LLT positions. Compared with the left LLT position, the right LLT position was associated with a significantly lower peak compression force during both the first minute (51.39 ± 15.20 kg vs. 55.65 ± 13.83 kg, *p* = 0.014) and the second minute (44.37 ± 14.50 kg vs. 52.20 ± 12.79 kg, *p* < 0.001).

**TABLE 3:** Force and pressure distribution in left lateral tilt chest compressions.

Force and pressure distribution		Right side (*n* = 44)	Left side (*n* = 44)	*P* value^β^
		Mean ± SD	Mean ± SD	
Peak force (kg)	1st minute	51.39 ± 15.20	55.65 ± 13.83	0.014^∗^
	2nd minute	44.37 ± 14.50	52.20 ± 12.79	< 0.001^∗^
	*P* value^α^	< 0.001^∗^	0.004^∗^	
Peak force difference between inferior–superior (kg)	1st minute	1.28 ± 14.97	7.89 ± 23.08	0.066
	2nd minute	2.91 ± 13.88	9.07 ± 21.24	0.045^∗^
	*p* value^α^	0.155	0.315	
Release force (kg)	1st minute	3.13 ± 2.72	1.27 ± 1.43	< 0.001^∗^
	2nd minute	2.26 ± 2.81	0.87 ± 1.46	0.001^∗^
	*p* value^α^	0.003^∗^	< 0.001^∗^	
Displacement of the center of pressure in left to right direction (cm)	1st minute	2.50 ± 2.43	1.28 ± 1.05	0.009^∗^
	2nd minute	2.14 ± 1.05	1.19 ± 1.04	< 0.001^∗^
	*p* value^α^	0.381	0.261	
Displacement of the center of pressure in superior to inferior direction (cm)	1st minute	1.69 ± 1.07	1.96 ± 1.48	0.403
	2nd minute	1.96 ± 1.22	1.77 ± 1.23	0.492
	*p* value^α^	0.146	0.274	
Peak pressure (kg/cm2)	Two minutes	2.03 ± 0.76	1.62 ± 0.57	0.001^∗^
Peak pressure difference between inferior–superior (kg/cm2)	Two minutes	0.00 ± 0.66	0.09 ± 0.67	0.632

^∗^Statistical differences.

^α^The comparison between the first second and the second under the same compression method.

^β^The comparison of different compression methods at the same time.

In contrast, the right LLT position demonstrated a significantly higher release force during the first minute (3.13 ± 2.72 kg vs. 1.27 ± 1.43 kg, *p* < 0.001) and the second minute (2.26 ± 2.81 kg vs. 0.87 ± 1.46 kg, *p* = 0.001), indicating less complete recoil. Moreover, both peak and release forces showed a significant decline from the first to the second minute in both positions, suggesting fatigue‐related degradation in compression quality over time.

### 3.4. Compression Center Shift and Palm Pressure Distribution (Table 3)

We also recorded whether the center position of the ECC will shift during the operation. Table 3 displays the results left–right and superior–inferior displacement and right LLT ECC has more left–right displacement than left LLT ECC in first minute (2.50 ± 2.43 cm vs. 1.28 ± 1.05 cm, *p* = 0.009) and second minute (2.14 ± 1.05 cm vs. 1.19 ± 1.04 cm, *p* < 0.001). There are no significantly differences in superior–inferior displacement. There is no statistical difference between the first and second minutes of the displacement.

Table 3 also shows the peak pressure and pressure distribution of the palm during LLT ECC. The right LLT position produced significantly higher peak palm pressure compared with the left LLT (2.03 ± 0.76 kg/cm^2^ vs. 1.62 ± 0.57 kg/cm^2^, *p* = 0.001). However, the difference in peak pressure across the inferior–superior axis was not statistically significant (0.00 ± 0.66 kg/cm^2^ vs. 0.09 ± 0.67 kg/cm^2^, *p* = 0.632).

### 3.5. Physiological Parameters Before and After ECC (Table 4)

Table 4 presents the SBP, DBP, and HR measured before and after LLT ECC. No significant differences in SBP, DBP, or HR were observed between the right and left LLT positions, either before or after compressions. Both HR and SBP increased significantly after each ECC (all *p* < 0.001). DBP showed no significant change.

**TABLE 4:** Physiological parameters before and after right‐side and left‐side left lateral tilt chest compressions.

Physiological parameters		Right side (*n* = 44)	Left side (*n* = 44)	*P* value^β^
		Mean ± SD	Mean ± SD	
Systolic blood pressure (mmHg)	Before	116.59 ± 15.14	118.11 ± 16.14	0.436
	After	128.93 ± 18.54	128.90 ± 19.25	0.989
	*P* value^α^	< 0.001^∗^	< 0.001^∗^	
Diastolic blood pressure (mmHg)	Before	75.50 ± 11.17	75.30 ± 10.17	0.851
	After	76.00 ± 9.79	77.32 ± 10.66	0.286
	*P* value^α^	0.613	0.137	
Heart rate (/min)	Before	78.64 ± 12.43	79.75 ± 11.11	0.417
	After	99.20 ± 16.77	98.48 ± 17.32	0.661
	*P* value^α^	< 0.001^∗^	< 0.001^∗^	

^∗^Statistical differences.

^α^The comparison between the first second and the second under the same compression method.

^β^The comparison of different compression methods at the same time.

### 3.6. Perceived Fatigue and Discomfort (Table 5)

Table 5 summarizes participants’ perceived fatigue and discomfort scores during chest compressions. Right‐sided LLT ECC was associated with significantly greater fatigue compared with the left side (5.82 ± 3.20 vs. 4.61 ± 3.01; *p* = 0.011). However, there were no statistically significant differences in reported hand discomfort (5.48 ± 2.61 vs. 4.64 ± 2.74; *p* = 0.062) or overall discomfort (5.79 ± 2.72 vs. 5.21 ± 2.15; *p* = 0.091) between the two positions.

**TABLE 5:** Fatigue and discomfort after right‐side and left‐side left lateral tilt chest compressions.

Fatigue and discomfort	Right side (*n* = 44)	Left side (*n* = 44)	*p* value
	Mean ± SD	Mean ± SD	
Fatigue (1–10)	5.82 ± 3.20	4.61 ± 3.01	0.011
Hand discomfort (1–10)	5.48 ± 2.61	4.64 ± 2.74	0.062
Overall discomfort (1–10)	5.79 ± 2.72	5.21 ± 2.15	0.091

## 4. Discussion

LLT has been shown to reduce uterine compression on the IVC and effectively increase blood return to the heart [20–23]. However, current guidelines do not recommend LLT ECC due to lack of evidence supporting its effect [33–36]. In our study, we found that the overall quality of LLT ECC was suboptimal, especially in the patient’s right side. Although the compression rate met the recommended standard of 100–120 compressions per minute in both positions and the hand placement did not significantly deviate from the correct site, other key components of high‐quality CPR were not achieved.

Specifically, compression depth and chest recoil were consistently inadequate in both left and right sided LLT ECC, with the right side performing worse. These findings suggest that LLT positioning, especially on the right side, may compromise the effectiveness of chest compressions and limit the delivery of high‐quality CPR.

### 4.1. Quality of LLT Chest Compressions

Our findings demonstrated that chest compression depth during LLT ECC was suboptimal on both sides, with only a small proportion of participants achieving the recommended 5‐6 cm depth. This is consistent with previous studies suggesting that LLT positioning may reduce compression effectiveness by altering force application and chest wall compliance [35, 41]. While left‐sided LLT ECC produced slightly better compression depth than right‐sided LLT ECC, neither position consistently met the depth criterion for HQCPR.

Despite the limitation in depth, both left‐ and right‐side LLT ECC maintain appropriate compression rates, consistent with ACLS recommendations [24, 25]. Rate consistency indicates that rescuers can effectively adjust their compression cadence at the LLT position, even if other parameters such as compression depth and recoil force are affected.

### 4.2. Forces Distribution and Pressure Analysis

Force analysis revealed that the left‐side LLT ECC generated higher peak compression force and lower residual release force compared with right‐side LLT ECC. This suggests that left‐side LLT ECC contributes to better compression recoil, which is an important component of HQCPR. Increased residual release force in the right‐side LLT ECC may result in incomplete recoil, limiting effective blood flow during cardiopulmonary resuscitation [35, 42, 43].

Furthermore, force decay was observed over time in both locations, indicating the onset of rescuer fatigue. The decrease in peak force and depth within 2 minutes highlights the importance of frequent rotation of rescuers to maintain effective ECC quality. Notably, a greater displacement of the pressure center was seen in right‐sided LLT ECC, suggesting decreased positional stability. This instability may further contribute to diminished compression quality and accelerate rescuer fatigue.

### 4.3. Physiological Impact on Rescuers

Physiological parameters including HR and BP increased following ECC, reflecting the expected physical exertion associated with chest compressions. Although perceived fatigue was higher in the right‐side LLT ECC, no significant difference in overall physical discomfort was observed between the two positions. These findings suggest that while left‐side LLT ECC may yield better quality, it does not impose a significantly greater physiological burden on the rescuer.

### 4.4. Clinical Implications and Guidelines Consideration

The results of this study align with the 2015 AHA guideline recommendations, which recommend manual uterine displacement over LLT due to concerns regarding the efficacy of ECC in LLT position. The observed reduction in compression depth and incomplete recoil in both LLT positions further supports this recommendation. Nevertheless, in scenarios with limited personnel, LLT remains a feasible alternative when manual uterine displacement is not possible.

The superior performance of left‐side LLT ECC suggests that if LLT is used, compression in the left side should be prioritized whenever possible. Additionally, given the overall limitations of compression depth, further research should explore alternative positioning strategies or assistive devices (e.g., bracing devices) to enhance the efficacy of compressions in pregnant patients.

### 4.5. Limitations

This study has several limitations. First, it was performed using a manikin model, which may not fully replicate the complex physiological changes and tissue resistance of the pregnant human body. Second, all participants were experienced healthcare providers with ACLS certification, which may not reflect the performance of less experienced rescuers. Finally, the study only evaluated a 2 minutes’ compression cycle, whereas real‐life resuscitation efforts typically last longer and involve rotations of rescuers. Future studies should investigate prolonged ECC performance, the impact of manual uterine transposition, and alternative positioning techniques for optimizing HQCPR in pregnant patients.

## 5. Conclusion

This study demonstrated that left‐side LLT ECC resulted in better compression depth and recoil compared with right‐side LLT ECC. However, neither technique consistently achieved the standards of HQCPR, particularly in achieving adequate compression depth. These findings support current AHA recommendations favoring manual uterine displacement over LLT. If LLT is used, left‐side LLT ECC is better than right‐side LLT ECC. Further research is needed to optimize the quality of CPR in maternal cardiac arrest situations to ensure optimal outcomes for mother and fetus.

## 6. Future Research

Based on this study, future research could examine the differences between normal position and LLT to understand whether LLT affects chest compression. Furthermore, the effectiveness of mechanical CPR device when used with LLT could be explored.

## Author Contributions

Chia‐Lung Kao: conceived and designed the experiments; performed the experiments; analyzed and interpreted the data; contributed reagents, materials, analysis tools, or data; and wrote the paper. Jui‐Yi Tsou: analyzed and interpreted the data and contributed reagents, materials, analysis tools, or data. Ming‐Yuan Hong and Chih‐Jan Chang performed the experiments. Fong‐Chin Su: conceived and designed the experiments. Chih‐Hsien Chi: conceived and designed the experiments; performed the experiments; contributed reagents, materials, analysis tools, or data; and wrote the paper.

## Funding

This study was partly supported by the Research Project (NCKUH‐10703058) of National Cheng Kung University Hospital.

## Ethics Statement

This study was approved by the Ethics Review Board of National Cheng Kung University Hospital (IRB: NCKUH B‐ER‐105–262).

## Conflicts of Interest

The authors declare no conflicts of interest.

## Data Availability

The datasets generated and analyzed during the current study are available from the corresponding author upon reasonable request.
